# Patient safety in remote primary care encounters: multimethod qualitative study combining Safety I and Safety II analysis

**DOI:** 10.1136/bmjqs-2023-016674

**Published:** 2023-11-28

**Authors:** Rebecca Payne, Aileen Clarke, Nadia Swann, Jackie van Dael, Natassia Brenman, Rebecca Rosen, Adam Mackridge, Lucy Moore, Asli Kalin, Emma Ladds, Nina Hemmings, Sarah Rybczynska-Bunt, Stuart Faulkner, Isabel Hanson, Sophie Spitters, Sietse Wieringa, Francesca H Dakin, Sara E Shaw, Joseph Wherton, Richard Byng, Laiba Husain, Trisha Greenhalgh

**Affiliations:** 1 Nuffield Department of Primary Care Health Sciences, University of Oxford, Oxford, UK; 2 Nuffield Trust, London, UK; 3 Betsi Cadwaladr University Health Board, Bangor, UK; 4 Peninsula Schools of Medicine and Dentistry, University of Plymouth, Plymouth, UK; 5 Wolfson Institute of Population Health, Queen Mary University of London, London, UK; 6 Sustainable Health Unit, University of Oslo, Oslo, Norway

**Keywords:** Primary care, Diagnostic errors, Safety culture, Qualitative research, Prehospital care

## Abstract

**Background:**

Triage and clinical consultations increasingly occur remotely. We aimed to learn why safety incidents occur in remote encounters and how to prevent them.

**Setting and sample:**

UK primary care. 95 safety incidents (complaints, settled indemnity claims and reports) involving remote interactions. Separately, 12 general practices followed 2021–2023.

**Methods:**

Multimethod qualitative study. We explored causes of real safety incidents retrospectively (‘Safety I’ analysis). In a prospective longitudinal study, we used interviews and ethnographic observation to produce individual, organisational and system-level explanations for why safety and near-miss incidents (rarely) occurred and why they did not occur more often (‘Safety II’ analysis). Data were analysed thematically. An interpretive synthesis of why safety incidents occur, and why they do not occur more often, was refined following member checking with safety experts and lived experience experts.

**Results:**

Safety incidents were characterised by inappropriate modality, poor rapport building, inadequate information gathering, limited clinical assessment, inappropriate pathway (eg, wrong algorithm) and inadequate attention to social circumstances. These resulted in missed, inaccurate or delayed diagnoses, underestimation of severity or urgency, delayed referral, incorrect or delayed treatment, poor safety netting and inadequate follow-up. Patients with complex pre-existing conditions, cardiac or abdominal emergencies, vague or generalised symptoms, safeguarding issues, failure to respond to previous treatment or difficulty communicating seemed especially vulnerable. General practices were facing resource constraints, understaffing and high demand. Triage and care pathways were complex, hard to navigate and involved multiple staff. In this context, patient safety often depended on individual staff taking initiative, speaking up or personalising solutions.

**Conclusion:**

While safety incidents are extremely rare in remote primary care, deaths and serious harms have resulted. We offer suggestions for patient, staff and system-level mitigations.

**WHAT IS ALREADY KNOWN ON THIS TOPIC**

- Safety incidents are extremely rare in primary care but they do happen. Concerns have been raised about the safety of remote triage and remote consultations.

**WHAT THIS STUDY ADDS**

- Rare safety incidents (involving death or serious harm) in remote encounters can be traced back to various clinical, communicative, technical and logistical causes. Telephone and video encounters in general practice are occurring in a high-risk (extremely busy and sometimes understaffed) context in which remote workflows may not be optimised. Front-line staff use creativity and judgement to help make care safer.

**HOW THIS STUDY MIGHT AFFECT RESEARCH, PRACTICE OR POLICY**

- As remote modalities become mainstreamed in primary care, staff should be trained in the upstream causes of safety incidents and how they can be mitigated. The subtle and creative ways in which front-line staff already contribute to safety culture should be recognised and supported.

## Introduction

In early 2020, remote triage and remote consultations (together, ‘remote encounters’), in which the patient is in a different physical location from the clinician or support staff member, were rapidly expanded as a safety measure in many countries because they eliminated the risk of transmitting COVID-19.^1–4^ But by mid-2021, remote encounters had begun to be depicted as potentially *unsafe* because they had come to be associated with stories of patient harm, including avoidable deaths and missed cancers.^5–8^

Providing triage and clinical care remotely is sometimes depicted as a partial solution to the system pressures facing primary healthcare in many countries,^9–11^ including rising levels of need or demand, the ongoing impact of the COVID-19 pandemic and workforce challenges (especially short-term or longer-term understaffing). In this context, remote encounters may be an important component of a mixed-modality health service when used appropriately alongside in-person contacts.^12 13^ But this begs the question of what ‘appropriate’ and ‘safe’ use of remote modalities in a primary care context is. Safety incidents (defined as ‘any unintended or unexpected incident which could have, or did, lead to harm for one or more patients receiving healthcare^14^’) are extremely rare in primary healthcare consultations generally,^15 16^ in-hours general practice telephone triage^17^ and out-of-hours primary care.^18^ But the recent widespread expansion of remote triage and remote consulting in primary care means that a wider range of patients and conditions are managed remotely, making it imperative to re-examine where the risks lie.

Theoretical approaches to safety in healthcare fall broadly into two traditions.^19^ ‘Safety I’ studies focus on what went wrong. Incident reports are analysed to identify ‘root causes’ and ‘safety gaps’, and recommendations are made to reduce the chance that further similar incidents will happen in the future.^20^ Such studies, undertaken in isolation, tend to lead to a tightening of rules, procedures and protocols. ‘Safety II’ studies focus on why, most of the time, things do not go wrong. Ethnography and other qualitative methods are employed to study how humans respond creatively to unique and unforeseen situations, thereby preventing safety incidents most of the time.^19^ Such studies tend to show that actions which achieve safety are highly context specific, may entail judiciously *breaking* the rules and require human qualities such as courage, initiative and adaptability.^21^ Few previous studies have combined both approaches.

In this study, we aimed to use Safety I methods to learn why safety incidents occur (although rarely) in remote primary care encounters and also apply Safety II methods to examine the kinds of creative actions taken by front-line staff that contribute to a safety culture and thereby *prevent* such incidents.

## Methods

### Study design and origins

Multimethod qualitative study across UK, including incident analysis, longitudinal ethnography and national stakeholder interviews.

The idea for this safety study began during a longitudinal ethnographic study of 12 general practices across England, Scotland and Wales as they introduced (and, in some cases, subsequently withdrew) various remote and digital modalities. Practices were selected for maximum diversity in geographical location, population served and digital maturity and followed from mid-2021 to end 2023 using staff and patient interviews and in-person ethnographic visits. The study protocol,^22^ baseline findings^23^ and a training needs analysis^24^ have been published. To provide context for our ethnography, we interviewed a sample of national stakeholders in remote and digital primary care, including out-of-hours providers running telephone-led services, and held four online multistakeholder workshops, one of which was on the theme of safety, for policymakers, clinicians, patients and other parties. Early data from this detailed qualitative work revealed staff and patient concerns about the safety of remote encounters but no actual examples of harm.

To explore the safety theme further, we decided to take a dual approach. First, following Safety I methodology for the study of rare harms,^20^ we set out to identify and analyse a sample of safety incidents involving remote encounters. These were sourced from arm’s-length bodies (NHS England, NHS Resolution, Healthcare Safety Investigation Branch) and providers of healthcare at scale (health boards, integrated care systems and telephone advice services), since our own small sample had not identified any of these rare occurrences. Second, we extended our longitudinal ethnographic design to more explicitly incorporate Safety II methodology,^19^ allowing us to examine safety culture and safety practices in our 12 participating general practices, especially the adaptive work done by staff to avert potential safety incidents.

### Data sources and management

Table 1 summarises the data sources.

**Table 1:** Summary of data sources

Source and type of data	Dates	Nature of full dataset	Subset of data analysed for this paper	Strengths	Limitations
**Safety incident dataset**					
Safety incidents from NHS England complaints	January 2021 to July 2022	Quarterly primary care complaints review slide sets, manually searched for remote encounters.	69 complaints relating to remotely delivered care (28 pages total).	Verified cases. Large numbers.	Skewed sample (only cases with complaints), and data preselected by NHS England, so unable to verify if representative of all such complaints. Safety issues which take years to emerge will have been missed.
Safety incidents from NHS Resolution claims	January 2015 to May 2023	Closed claims with an incident description which included at least one of telephone, phone, online, remote or triage. 22 cases identified.	16 cases included (160 pages total) (6 cases excluded, 3 because the complaint was unrelated to a remote consultation, and 3 due to insufficient information).	In-depth information on cases, often containing clinical records and local investigations such as RCAs.	Skewed sample (rare cases involving death or serious harm). Including only closed cases means some occurred several years ago. Complex cases may take longer to reach closure.
Safety incidents from investigation into COVID-19 telephone advice^7^	Mostly March to June 2020; also considered later COVID peaks in 2021/2022	National investigation by the Healthcare Safety Investigation Branch into set-up, design and delivery of COVID-19 telephone triage service accessed via 111 and into the patient pathway delivered by 111.	Full investigation report (86 pages; 4 cases highlighted as exemplars of wider issues).	In-depth investigation and analysis of a specific safety period in the early months of the pandemic.	Cases only reflect the earliest period of the pandemic (which was highly atypical) and are skewed towards the most severe examples.
Safety incidents from NHS 111	2021–2023	List of safety events relating to remote consulting in NHS 111 Wales with case examples provided by a senior clinician after a stakeholder interview.	List of key themes in patient safety, illustrated by fictionalised cases (2 pages; 6 cases).	National provider; large number of remote consultations, allowing rare cases to be identified.	Small number of cases. Procedures and systems for out-of-hours telephone provider differ from other parts of primary care.
**Safety culture and safety practices dataset**					
Multisite longitudinal case study of remote care in general practice^23^ ^22^	September 2021 to August 2023	12 general practices in England, Wales and Scotland followed for 24 months. Ethnography, staff and patient interviews and documents such as websites and leaflets. Data transcribed and coded on NVivo.	Fieldnotes and interviews where staff had highlighted safety concerns, fieldnotes relating to safety issues (30 pages total). Includes in-depth ethnographic observation of reception areas where decisions about mode of consultation are made.	Detailed ethnographic material; diverse sample. Insights into how and why staff make situated judgements and ‘break the rules’ to protect patient safety.	Practice-level information unlikely to capture rare events that may only be detected at scale.
Interviews with out-of-hours providers and trainers	2022	Interviews with 8 clinical leads working out of hours or in NHS 111 (default modality telephone) in England, Scotland and Wales.	Extracts from interviews and training materials shared by these providers (20 pages total).	Insights into a system where the default modality is telephone and the service has years of experience and an established system of supervision and training.	Out-of-hours primary care is not directly comparable to in-hours general practice.
Multistakeholder workshop	September 2022	Online workshop with 61 participants including clinicians, national clinical leads, representatives from arm’s-length bodies, practice staff and laypeople. Plenaries and breakout groups videotaped.	Interdisciplinary group discussions about safety, presentation by the 111 COVID Clinical Assessment Service, South Central Ambulance Service. 4 hours of video footage (11 pages of transcribed extracts).	Very rich and nuanced discussions among large number of participants across multiple sectors. Breakout groups allowed a wide range of views to be captured.	Clinical details of cases mentioned in discussions could not be verified (hence were not included in our sample of safety incidents).
Interviews with GP trainers and trainees	2023	Interviews with 10 GP trainers and 10 GP registrars who had trained during the pandemic, undertaken by recently trained GP.	Trainers’ comments on trainees’ ability to assess clinical risk in the remote environment; registrars’ perceptions on their own confidence in this regard.	Insights into how the rapid shift to remote had compromised training and supervision of clinicians.	Sample only included GPs; study was not resourced to do similar studies on other clinicians.
Expert interview and sense check of draft findings with national stakeholders	May to June 2023	Stakeholders in strategic safety roles and subject matter experts sampled from arm’s-length bodies, government and health boards.	Extracts of data from 6 interviews including 3 representatives of arm’s-length bodies, 1 Department of Health, 2 health boards (12 pages total).	Informants worked at senior levels with an oversight at system level. Included representation from 111/OOH expert familiar with remote consults.	Risk of bias in recall.

GP, general practitioner; OOH, out of hours; RCAs, root cause analyses.

The Safety I dataset (rows 2-5) consisted of 95 specific incident reports, including complaints submitted to the main arm’s-length NHS body in England, NHS England, between 2020 and 2023 (n=69), closed indemnity claims that had been submitted to a national indemnity body, NHS Resolution, between 2015 and 2023 (n=16), reports from an urgent care telephone service in Wales (NHS 111 Wales) between 2020 and 2023 (n=6) and a report on an investigation of telephone advice during the COVID-19 crisis between 2020 and 2022^7^ (n=4). These 95 incidents were organised using Microsoft Excel spreadsheets.

The Safety II dataset (rows 6-10) consisted of extracts from fieldnotes, workshop transcripts and interviews collected over 2 years, stored and coded on NVivo qualitative software. These were identified by searching for text words and codes (e.g. ‘risk’, ‘safety’, ‘incident’) and by asking researchers-in-residence, who were closely familiar with practices, to highlight safety incidents involving harm and examples of safety-conscious work practices. This dataset included over 100 formal interviews and numerous on-the-job interviews with practice staff, plus interviews with a sample of 10 GP (general practitioner) trainers and 10 GP trainees (penultimate row of table 1) and with six clinical safety experts identified through purposive sampling from government, arm’s-length bodies and health boards (bottom row of table 1).

### Data analysis

We analysed incident reports, interview data and ethnographic fieldnotes using thematic analysis as described by Braun and Clarke.^25^ These authors define a theme as an important, broad pattern in a set of qualitative data, which can (where necessary) be further refined using coding.

Themes in the incident dataset were identified by five steps. First, two researchers (both medically qualified) read each source repeatedly to gain familiarity. Second, those researchers worked independently using Braun and Clarke’s criterion (‘whether it captures something important in relation to the overall research question’—p 82^25^) to identify themes. Third, they discussed their initial interpretations with each other and resolved differences through discussion. Fourth, they extracted evidence from the data sources to illustrate and refine each theme. Finally, they presented their list of themes along with illustrative examples to the wider team. Cases used to illustrate themes were systematically fictionalised by changing age, randomly allocating gender and altering clinical details.^26^ For example, an acute appendicitis could be changed to acute diverticulitis if the issue was a missed acute abdomen.

These safety themes were then used to sensitise us to seek relevant (confirming and disconfirming) material from our ethnographic and interview datasets. For example, the theme ‘poor communication’ (and subthemes such as ‘failure to seek further clarification’ within this) promoted us to look for examples in our stakeholder interviews of poor communication offered as a cause of safety incidents and examples in our ethnographic notes of *good* communication (including someone seeking clarification). We used these wider data to add nuance to the initial list of themes.

As a final sense-checking step, the draft findings from this study were shown to each of the six safety experts in our sample and refined in the light of their comments (in some cases, for example, they considered the case to have been overfictionalised, thereby losing key clinical messages; they also gave additional examples to illustrate some of the themes we had identified, which underlined the importance of those themes).

## Results

### Overview of dataset

The dataset (table 1) consisted of 95 incident reports (see fictionalised examples in box 1), plus approximately 400 pages of extracts from interviews, ethnographic fieldnotes and workshop discussions, including situated safety practices (see examples in box 2), plus strategic insights relating to policy, organisation and planning of services. Notably, almost all incidents related to telephone calls.

Box 1 **Examples of safety incidents involving death or serious harm in remote encounters**

All these cases have been systematically fictionalised as explained in the text.

**Case 1 (death)**

A woman in her 70s experiencing sudden breathlessness called her GP (general practitioner) surgery. The receptionist answered the phone and informed her that she would place her on the doctor’s list for an emergency call-back. The receptionist was distracted by a patient in the waiting room and did not do so. The patient deteriorated and died at home that afternoon.—NHS Resolution case, pre-2020

**Case 2 (death)**

An elderly woman contacted her GP after a telephone contact with the out-of-hours service, where constipation had been diagnosed. The GP prescribed laxatives without seeing the patient. The patient self-presented to the emergency department (ED) the following day in obstruction secondary to an incarcerated hernia and died in the operating theatre.—NHS Resolution case, pre-2020

**Case 3 (risk to vulnerable patients)**

A daughter complained that her elderly father was unable to access his GP surgery as he could not navigate the online triage system. When he phoned the surgery directly, he was directed back to the online system and told to get a relative to complete the form for him.—Complaint to NHS England, 2021

**Case 4 (harm)**

A woman in her first pregnancy at 28 weeks’ gestation experiencing urinary incontinence called NHS 111. She was taken down by a ‘urinary problems’ algorithm. Both the call handler and the subsequent clinician failed to recognise that she had experienced premature rupture of membranes. She later presented to the maternity department in active labour, and the opportunity to give early steroids to the premature infant was missed.—NHS Resolution case, pre-2020

**Case 5 (death)**

A doctor called about a 16-year-old girl with lethargy, shaking, fever and poor oral intake who had been unwell for 5 days. The doctor spoke to her older sister and advised that the child had likely glandular fever and should rest. When the parents arrived home, they called an ambulance but the child died of sepsis in the ED.—NHS Resolution case, pre-2020

**Case 6 (death)**

A 40-year-old woman, 6 weeks after caesarean section, contacted her GP due to shortness of breath, increased heart rate and dry cough. She was advised to get a COVID test and to dial 111 if she developed a productive cough, fever or pain. The following day she collapsed and died at home. The postmortem revealed a large pulmonary embolus. On reviewing the case, her GP surgery felt that had she been seen face to face, her oxygen saturations would have been measured and may have led to suspicion of the diagnosis.—NHS Resolution case, 2020

**Case 7 (death)**

A son complained that his father with diabetes and chronic kidney disease did not receive any in-person appointments over a period of 1 year. His father went on to die following a leg amputation arising from a complication of his diabetes.—Complaint to NHS England, 2021

**Case 8 (death)**

A 73-year-old diabetic woman with throat pain and fatigue called the surgery. She was diagnosed with a viral illness and given self-care advice. Over the next few days, she developed worsening breathlessness and was advised to do a COVID test and was given a pulse oximeter. She was found dead at home 4 days later. Postmortem found a blocked coronary artery and a large amount of pulmonary oedema. The cause of death was myocardial infarction and heart failure.—NHS Resolution case, pre-2020

**Case 9 (harm)**

A patient with a history of successfully treated cervical cancer developed vaginal bleeding. A diagnosis of fibroids was made and the patient received routine care by telephone over the next few months until a scan revealed a local recurrence of the original cancer.—Complaint to NHS England, 2020

**Case 10 (death)**

A 65-year-old female smoker with chronic cough and breathlessness presented to her GP. She was diagnosed with chronic obstructive pulmonary disease (COPD) and monitored via telephone. She did not respond to inhalers or antibiotics but continued to receive telephone monitoring without further investigation. Her symptoms continued to worsen and she called an ambulance. In the ED, she was diagnosed with heart failure and died soon after.—Complaint to NHS England, 2021

**Case 11 (harm)**

A 30-year-old woman presented with intermittent episodes of severe dysuria over a period of 2 years. She was given repeated courses of antibiotics but no urine was sent for culture and she was not examined. After 4 months of symptoms, she saw a private GP and was diagnosed with genital herpes.—Complaint to NHS England, 2021

**Case 12 (harm)**

There were repeated telephone consultations about a baby whose parents were concerned that the child was having a funny colour when feeding or crying. The 6-week check was done by telephone and at no stage was the child seen in person. Photos were sent in, but the child’s dark skin colour meant that cyanosis was not easily apparent to the reviewing clinician. The child was subsequently admitted by emergency ambulance where a significant congenital cardiac abnormality was found.—Complaint to NHS England, 2020^1^

**Case 13 (harm)**

A 35-year-old woman in her third trimester of pregnancy had a telephone appointment with her GP about a breast lump. She was informed that this was likely due to antenatal breast changes and was not offered an in-person appointment. She attended after delivery and was referred to a breast clinic where a cancer was diagnosed.—Complaint to NHS England, 2020

**Case 14 (harm)**

A 63-year-old woman with a variety of physical symptoms including diarrhoea, hip girdle pain, palpitations, light-headedness and insomnia called her surgery on multiple occasions. She was told her symptoms were likely due to anxiety, but was diagnosed with stage 4 ovarian cancer and died soon after.—Complaint to NHS England, 2021

**Case 15 (death)**

A man with COPD with worsening shortness of breath called his GP surgery. The staff asked him if it was an emergency, and when the patient said no, scheduled him for 2 weeks later. The patient died before the appointment.—Complaint to NHS England, 2021

Box 2 **Examples of safety practices**

**Case 16 (safety incident averted by switching to video call for a sick child)**

‘I’ve remembered one father that called up. Really didn’t seem to be too concerned. And was very much under-playing it and then when I did a video call, you know this child… had intercostal recession… looked really, really poorly. And it was quite scary actually that, you know, you’d had the conversation and if you’d just listened to what Dad was saying, actually, you probably wouldn’t be concerned.’—GP (general practitioner) interview 2022

**Case 17 (‘red flag’ spotted by support staff member)**

A receptionist was processing routine ‘administrative’ encounters sent in by patients using AccuRx (text messaging software). She became concerned about a sick note renewal request from a patient with a mental health condition. The free text included a reference to feeling suicidal, so the receptionist moved the request to the ‘red’ (urgent call-back) list. In interviews with staff, it became apparent that there had recently been heated discussion in the practice about whether support staff were adding ‘too many’ patients to the red list. After discussing cases, the doctors concluded that it should be them, not the support staff, who should absorb the risk in uncertain cases. The receptionist said that they had been told: ‘if in doubt, put it down as urgent and then the duty doctor can make a decision.’—Ethnographic fieldnotes from general practice 2023

**Case 18 (‘check-in’ phone call added on busy day)**

A duty doctor was working through a very busy Monday morning ‘urgent’ list. One patient had acute abdominal pain, which would normally have triggered an in-person appointment, but there were no slots and hard decisions were being made. This patient had had the pain already for a week, so the doctor judged that the general rule of in-person examination could probably be over-ridden. But instead of simply allocating to a call-back, the doctor asked a support staff member to phone the patient, ask ‘are you OK to wait until tomorrow?’ and offer basic safety-netting advice.—Ethnographic fieldnotes from general practice 2023

**Case 19 (receptionist advocating on behalf of ‘angry’ walk-in patient)**

A young Afghan man with limited English walked into a GP surgery on a very busy day, ignoring the prevailing policy of ‘total triage’ (make contact by phone or online in the first instance). He indicated that he wanted a same-day in-person appointment for a problem he perceived as urgent. A heated exchange occurred with the first receptionist, and the patient accused her of ‘racism’. A second receptionist of non-white ethnicity herself noted the man’s distress and suspected that there may indeed be an urgent problem. She asked the first receptionist to leave the scene, saying she wanted to ‘have a chat’ with the patient (‘the colour of my skin probably calmed him down more than anything’). Through talking to the patient and looking through his record, she ascertained that he had an acute infection that likely needed prompt attention. She tried to ‘bend the rules’ and persuade the duty doctor to see the patient, conveying the clinical information but deliberately omitting the altercation. But the first receptionist complained to the doctor (‘he called us racists’) and the doctor decided that the patient would not therefore be offered a same-day appointment. The second receptionist challenged the doctor (‘that’s not a reason to block him from getting care’). At this point, the patient cried and the second receptionist also became upset (‘this must be serious, you know’). On this occasion, despite her advocacy the patient was not given an immediate appointment.—Ethnographic fieldnotes from general practice 2022

**Case 20 (long-term condition nurse visits ‘unengaged’ patients at home)**

An advanced nurse practitioner talks of two older patients, each with a long-term condition, who are ‘unengaged’ and lacking a telephone. In this practice, all long-term condition reviews are routinely done by phone. She reflects that some people ‘choose not to have avenues of communication’ (ie, are *deliberately* not contactable), and that there may be reasons for this (‘maybe health anxiety or just old’). She has, on occasion, ‘turned up’ unannounced at the patient’s home and asked to come in and do the review, including bloods and other tests. She reflects that while most patients engage well with the service, ‘half my job is these patients who don’t engage very well.’—Ethnographic fieldnotes from digitally advanced general practice 2022

**Case 21 (doctor over-riding patient’s request for telephone prescribing)**

A GP trainee described a case of a 53-year-old first-generation immigrant from Pakistan, a known smoker with hypertension and diabetes. He had booked a telephone call for vomiting and sinus pain. There was no interpreter available but the man spoke some English. He said he had awoken in the night with pain in his sinuses and vomiting. All he wanted was painkillers for his sinuses. The story did not quite make sense, and the man ‘sounded unwell’. The GP told him he needed to come in and be examined. The patient initially resisted but was persuaded to come in. When the GP went to call him in, the man was visibly unwell and lying down in the waiting room. When seen in person, he admitted to shoulder pain. The GP sent him to accident and emergency (A&E) where a myocardial infarction was diagnosed.—Trainee interview 2023

Below, we describe the main themes that were evident in the safety incidents: a challenging organisational and system context, poor communication compounded by remote modalities, limited clinical information, patient and carer burden and inadequate training. Many safety incidents illustrated multiple themes—for example, poor communication *and* failures of clinical assessment or judgement *and* patient complexity *and* system pressures. In the detailed findings below, we illustrate why safety incidents occasionally occur and why they are usually avoided.

### The context for remote consultations: system and operational challenges

Introduction of remote triage and expansion of remote consultations in UK primary care occurred at a time of unprecedented system stress (an understaffed and chronically under-resourced primary care sector, attempting to cope with a pandemic).^23^ Many organisations had insufficient telephone lines or call handlers, so patients struggled to access services (eg, half of all calls to the emergency COVID-19 telephone service in March 2020 were never answered^7^). Most remote consultations were by telephone.^27^

Our safety incident dataset included examples of technically complex access routes which patients found difficult or impossible to navigate (case 3 in box 1) and which required non-clinical staff to make clinical or clinically related judgements (cases 4 and 15). Our ethnographic dataset contained examples of inflexible application of triage rules (eg, no face-to-face consultation unless the patient had already had a telephone call), though in other practices these rules could be over-ridden by staff using their judgement or asking colleagues. Some practices had a high rate of failed telephone call-backs (patient unobtainable).

High demand, staff shortages and high turnover of clinical and support staff made the context for remote encounters inherently risky. Several incidents were linked to a busy staff member becoming distracted (case 1). Telephone consultations, which tend to be shorter, were sometimes used in the hope of improving efficiency. Some safety incidents suggested perfunctory and transactional telephone consultations, with flawed decisions made on the basis of incomplete information (eg, case 2).

Many practices had shifted—at least to some extent—from a demand-driven system (in which every request for an appointment was met) to a capacity-driven one (in which, if a set capacity was exceeded, patients were advised to seek care elsewhere), though the latter was often used flexibly rather than rigidly with an expectation that some patients would be ‘squeezed in’. In some practices, capacity limits had been introduced to respond to escalation of demand linked to overuse of triage templates (eg, to inquire about minor symptoms).

As a result of task redistribution and new staff roles, a single episode of care for one problem often involved multiple encounters or tasks distributed among clinical and non-clinical staff (often in different locations and sometimes also across in-hours and out-of-hours providers). Capacity constraints in onward services placed pressure on primary care to manage risk in the community, leading in some cases to failure to escalate care appropriately (case 6).

Some safety incidents were linked to organisational routines that had not adapted sufficiently to remote—for example, a prescription might be issued but (for various reasons) it could not be transmitted electronically to the pharmacy. Certain urgent referrals were delayed if the consultation occurred remotely (a referral for suspected colon cancer, for example, would not be accepted without a faecal immunochemical test).

Training, supervising and inducting staff was more difficult when many were working remotely. If teams saw each other less frequently, relationship-building encounters and ‘corridor’ conversations were reduced, with knock-on impacts for individual and team learning and patient care. Those supervising trainees or allied professionals reported loss of non-verbal cues (eg, more difficult to assess how confident or distressed the trainee was).

Clinical and support staff regularly used initiative and situated judgement to compensate for an overall lack of system resilience (box 1). Many practices had introduced additional safety measures such as lists of patients who, while not obviously urgent, needed timely review by a clinician. Case 17 illustrates how a rule of thumb ‘if in doubt, put it down as urgent’ was introduced and then applied to avert a potentially serious mental health outcome. Case 18 illustrates how, in the context of insufficient in-person slots to accommodate all high-risk cases, a unique safety-netting measure was customised for a patient.

### Poor communication is compounded by remote modalities

Because sense data (eg, sight, touch, smell) are missing,^28^ remote consultations rely heavily on the history. Many safety incidents were characterised by insufficient or inaccurate information for various reasons. Sometimes (cases 2, 5, 6, 8, 9, 10 and 11), the telephone consultation was too short to do justice to the problem; the clinician asked few or no questions to build rapport, obtain a full history, probe the patient’s answers for additional detail, confirm or exclude associated symptoms and inquire about comorbidities and medication. Video provided some visual cues but these were often limited to head and shoulders, and photographs were sometimes of poor quality.

Cases 2, 4, 5 and 9 illustrate the dangers of relying on information provided by a third party (another staff member or a relative). A key omission (eg, in case 5) was failing to ask why the patient was unable to come to the phone or answer questions directly.

Some remote triage conversations were conducted using an inappropriate algorithm. In case 4, for example, the call handler accepted a pregnant patient’s assumption that leaking fluid was urine when the problem was actually ruptured membranes. The wrong pathway was selected; vital questions remained unasked; and a skewed history was passed to (and accepted by) the clinician. In case 8, the patient’s complaint of ‘throat’ pain was taken literally and led to ‘viral illness’ advice, overlooking a myocardial infarction.

The cases in box 2 illustrate how staff compensated for communication challenges. In case 16, a GP plays a hunch that a father’s account of his child’s asthma may be inaccurate and converts a phone encounter to video, revealing the child’s respiratory distress. In case 19 (an in-person encounter but relevant because the altercation occurs partly because *remote* triage is the default modality), one receptionist correctly surmises that the patient’s angry demeanour may indicate urgency and uses her initiative and interpersonal skills to obtain additional clinical information. In case 20, a long-term condition nurse develops a labour-intensive workaround to overcome her elderly patients’ ‘lack of engagement’. More generally, we observed numerous examples of staff using both formal tools (eg, see ‘red list’ in case 17) and informal measures (eg, corridor chats) to pass on what they believed to be crucial information.

### Remote consulting can provide limited clinical information

Cases 2 and 4–14 all describe serious conditions including congenital cyanotic heart disease, pulmonary oedema, sepsis, cancer and diabetic foot which would likely have been readily diagnosed with an in-person examination. While patients often uploaded still images of skin lesions, these were not always of sufficient quality to make a confident diagnosis.

Several safety incidents involved clinicians assuming that a diagnosis made on a remote consultation was definitive rather than provisional. Especially when subsequent consultations were remote, such errors could become ingrained, leading to diagnostic overshadowing and missed or delayed diagnosis (cases 2, 8, 9, 10, 11 and 13). Patients with pre-existing conditions (especially if multiple or progressive), the very young and the elderly were particularly difficult to assess by telephone (cases 1, 2, 8, 10, 12 and 16). Clinical conditions difficult to assess remotely included possible cardiac pain (case 8), acute abdomen (case 2), breathing difficulties (cases 1, 6 and 10), vague and generalised symptoms (cases 5 and 14) and symptoms which progressed despite treatment (cases 9, 10 and 11). All these categories came up repeatedly in interviews and workshops as clinically risky.

Subtle aspects of the consultation which may have contributed to safety incidents in a telephone consultation included the inability to fully appraise the patient’s overall health and well-being (including indicators relevant to mental health such as affect, eye contact, personal hygiene and evidence of self-harm), general demeanour, level of agitation and concern, and clues such as walking speed and gait (cases 2, 5, 6, 7, 8, 10, 12 and 14). Our interviews included stories of missed cases of new-onset frailty and dementia in elderly patients assessed by telephone.

In most practices we studied, most long-term condition management was undertaken by telephone. This may be appropriate (and indeed welcome) when the patient is well and confident and a physical examination is not needed. But diabetes reviews, for example, require foot examination. Case 7 describes the deterioration and death of a patient with diabetes whose routine check-ups had been entirely by telephone. We also heard stories of delayed diagnosis of new diabetes in children when an initial telephone assessment failed to pick up lethargy, weight loss and smell of ketones, and point-of-care tests of blood or urine were not possible.

Nurses observed that remote consultations limit opportunities for demonstrating or checking the patient’s technique in using a device for monitoring or treating their condition such as an inhaler, oximeter or blood pressure machine.

Safety netting was inadequate in many remote safety incidents, even when provided by a clinician (cases 2, 5, 6, 8, 10, 12 and 13) but especially when conveyed by a non-clinician (case 15). Expert interviewees identified that making life-changing diagnoses remotely and starting patients on long-term medication without an in-person appointment was also risky.

Our ethnographic data showed that various measures were used to compensate for limited clinical information, including converting a phone consultation to video (case 16), asking the patient if they felt they could wait until an in-person slot was available (case 18), visiting the patient at home (case 20) and enacting a ‘if the history doesn’t make sense, bring the patient in for an in-person assessment’ rule of thumb (case 21). Out-of-hours providers added examples of rules of thumb that their services had developed over years of providing remote services, including ‘see a child face-to-face if the parent rings back’, ‘be cautious about third-party histories’, ‘visit a palliative care patient before starting a syringe driver’ and ‘do not assess abdominal pain remotely’.

### Remote modalities place additional burdens on patients and carers

Given the greater importance of the history in remote consultations, patients who lacked the ability to communicate and respond in line with clinicians’ expectations were at a significant disadvantage. Several safety incidents were linked to patients’ limited fluency in the language and culture of the clinician or to specific vulnerabilities such as learning disability, cognitive impairment, hearing impairment or neurodiversity. Those with complex medical histories and comorbidities, and those with inadequate technical set-up and skills (case 3), faced additional challenges.

In many practices, in-person appointments were strictly limited according to more or less rigid triage criteria. Some patients were unable to answer the question ‘is this an emergency?’ correctly, leading to their condition being deprioritised (case 15). Some had learnt to ‘game’ the triage system (eg, online templates^29^) by adapting their story to obtain the in-person appointment they felt they needed. This could create distrust and lead to inaccurate information on the patient record.

Our ethnographic dataset contained many examples of clinical and support staff using initiative to compensate for vulnerable patients’ inability or unwillingness to take on the additional burden of remote modalities (cases 19 and 20 in Box 2 30 31).

### Training for remote encounters is often inadequate

Safety incidents highlighted various training needs for support staff members (eg, customer care skills, risks of making clinical judgements) and clinicians (eg, limitations of different modalities, risks of diagnostic overshadowing). Whereas out-of-hours providers gave thorough training to novice GPs (covering such things as attentiveness, rapport building, history taking, probing, attending to contextual cues and safety netting) in telephone consultations,^32–34^ many in-hours clinicians had never been formally taught to consult by telephone. Case 17 illustrates how on-the-job training based on acknowledgement of contextual pressures and judicious use of rules of thumb may be very effective in averting safety incidents.

## Discussion

### Statement of principal findings

An important overall finding from this study is that examples of deaths or serious harms associated with remote encounters in primary care were extremely rare, amounting to fewer than 100 despite an extensive search going back several years.

Analysis of these 95 safety incidents, drawn from multiple complementary sources, along with rich qualitative data from ethnography, interviews and workshops has clarified where the key risks lie in remote primary care. Remote triage and consultations expanded rapidly in the context of the COVID-19 crisis; they were occurring in the context of resource constraints, understaffing and high demand. Triage and care pathways were complex, multilayered and hard to navigate; some involved distributed work among multiple clinical and non-clinical staff. In some cases, multiple remote encounters preceded (and delayed) a needed in-person assessment.

In this high-risk context, safety incidents involving death or serious harm were rare, but those that occurred were characterised by a combination of inappropriate choice of modality, poor rapport building, inadequate information gathering, limited clinical assessment, inappropriate clinical pathway (eg, wrong algorithm) and failure to take account of social circumstances. These led to missed, inaccurate or delayed diagnoses, underestimation of severity or urgency, delayed referral, incorrect or delayed treatment, poor safety netting and inadequate follow-up. Patients with complex or multiple pre-existing conditions, cardiac or abdominal emergencies, vague or generalised symptoms, safeguarding issues and failure to respond to previous treatment, and those who (for any reason) had difficulty communicating, seemed particularly at risk.

### Strengths and limitations of the study

The main strength of this study was that it combined the largest Safety I study undertaken to date of safety incidents in remote primary care (using datasets which have not previously been tapped for research), with a large, UK-wide ethnographic Safety II analysis of general practice as well as stakeholder interviews and workshops. Limitations of the safety incident sample (see final column in table 1) include that it was skewed towards very rare cases of death and serious harm, with relatively few opportunities for learning that did not result in serious harm. Most sources were retrospective and may have suffered from biases in documentation and recall. We also failed to obtain examples of safeguarding incidents (which would likely turn up in social care audits). While all cases involved a remote modality (or a patient who would not or could not use one), it is impossible to definitively attribute the harm to that modality.

### Comparison with existing literature

This study has affirmed previous findings that processes, workflows and training in in-hours general practice have not adapted adequately to the booking, delivery and follow-up of remote consultations.^24 35 36^ Safety issues can arise, for example, from how the remote consultation interfaces with other key practice routines (eg, for making urgent referrals for possible cancer). The sheer complexity and fragmentation of much remote and digital work underscores the findings from a systematic review of the importance of relational coordination (defined as *‘a mutually reinforcing process of communicating and relating for the purpose of task integration*’ (p 3)^37^) and psychological safety (defined as *‘people’s perceptions of the consequences of taking interpersonal risks in a particular context such as a workplace*’ (p 23)^38^) in building organisational resilience and assuring safety.

The additional workload and complexity associated with running remote appointments alongside in-person ones is cognitively demanding for staff and requires additional skills for which not all are adequately trained.^24 39 40^ We have written separately about the loss of traditional continuity of care as primary care services become digitised,^41–43^ and about the unmet training needs of both clinical and support staff for managing remote and digital encounters.^24^

Our findings also resonate with research showing that remote modalities can interfere with communicative tasks such as rapport building, establishing a therapeutic relationship and identifying non-verbal cues such as tearfulness^35 36 44^; that remote consultations tend to be shorter and feature less discussion, information gathering and safety netting^45–48^; and that clinical assessment in remote encounters may be challenging,^27 49 50^ especially when physical examination is needed.^35 36 51^ These factors may rarely contribute to incorrect or delayed diagnoses, underestimation of the seriousness or urgency of a case, and failure to identify a deteriorating trajectory.^35 36 52–54^

Even when systems seem adequate, patients may struggle to navigate them.^23 30 31^ This finding aligns with an important recent review of cognitive load theory in the context of remote and digital health services: because such services are more cognitively demanding for patients, they may widen inequities of access.^55^ Some patients lack navigating and negotiating skills, access to key technologies^13 36^ or confidence in using them.^30 35^ The remote encounter may require the patient to have a sophisticated understanding of access and cross-referral pathways, interpret their own symptoms (including making judgements about severity and urgency), obtain and use self-monitoring technologies (such as a blood pressure machine or oximeter) and convey these data in medically meaningful ways (eg, by completing algorithmic triage forms or via a telephone conversation).^30 56^ Furthermore, the remote environment may afford fewer opportunities for holistically evaluating, supporting or safeguarding the vulnerable patient, leading to widening inequities.^13 35 57^ Previous work has also shown that patients with pre-existing illness, complex comorbidities or high-risk states,^58 59^ language non-concordance,^13 35^ inability to describe their symptoms (eg, due to autism^60^), extremes of age^61^ and those with low health or system literacy^30^ are more difficult to assess remotely.

### Lessons for safer care

Many of the contributory factors to safety incidents in remote encounters have been suggested previously,^35 36^ and align broadly with factors that explain safety incidents more generally.^53 62 63^ This new study has systematically traced how upstream factors may, very rarely, combine to contribute to avoidable human tragedies—and also how primary care teams develop local safety practices and cultures to help avoid them. Our study provides some important messages for practices and policymakers.

First, remote encounters in general practice are mostly occurring in a system designed for in-person encounters, so processes and workflows may work less well.

Second, because the remote encounter depends more on history taking and dialogue, verbal communication is even more mission critical. Working remotely under system pressures and optimising verbal communication should both be priorities for staff training.

Third, the remote environment may increase existing inequities as patients’ various vulnerabilities (eg, extremes of age, poverty, language and literacy barriers, comorbidities) make remote communication and assessment more difficult. Our study has revealed impressive efforts from staff to overcome these inequities on an individual basis; some of these workarounds may become normalised and increase efficiency, but others are labour intensive and not scalable.

A final message from this study is that clinical assessment provides less information when a physical examination (and even a basic visual overview) is not possible. Hence, the remote consultation has a higher degree of inherent uncertainty. Even when processes have been optimised (eg, using high-quality triage to allocate modality), but especially when they have not, diagnoses and assessments of severity or urgency should be treated as more provisional and revisited accordingly. We have given examples in the Results section of how local adaptation and rule breaking bring flexibility into the system and may become normalised over time, leading to the creation of locally understood ‘rules of thumb’ which increase safety.

Overall, these findings underscore the need to share learning and develop guidance about the drivers of risk, how these play out in different kinds of remote encounters and how to develop and strengthen Safety II approaches to mitigate those risks. Table 2 shows proposed mitigations at staff, process and system levels, as well as a preliminary list of suggestions for patients, which could be refined with patient input using codesign methods.^64^

**Table 2:** Reducing safety incidents in remote primary care

Clinical conditions for which an in-person assessment is often required.	Acute chest or abdominal pain. Breathing difficulties. Breast lump. Palliative care. Physical injury. New psychosis. Diabetes reviews where eye or foot examination is needed. Persistent or progressive skin lesion. Acute history that does not make sense.
Clinical trajectories for which an in-person assessment is often required.	Condition has not resolved as expected (or has progressed) after previous remote consultation(s). Escalating parental concern. Acute condition overlaid on pre-existing complex illness (including mental health).
Patient-level features that make remote assessment more difficult and suggest a lower threshold for defaulting to in person.	Extremes of age. Care home residents if on-site staff not confident to undertake observations. Language non-concordance. Relevant impairment (eg, deafness). Conditions that may complicate communication (eg, autism). Low health literacy or system literacy. Lacks key technologies or the ability to use them.
Key features of effective safety netting.	Make clear to patient what the next steps in their care are, what to do if things get worse and action to take if expected care (eg, a call-back) does not happen. Make all points explicit; do not assume that the patient already knows. Fully document what safety-netting advice has been given. Back up verbal advice with text or email, including leaflet or web link if appropriate. Avoid rigid protocols and overscripting (but if non-clinicians are giving safety-netting advice, consider some basic standard scripts). Ask patient/family member/carer to repeat back safety-netting instructions.
Organisational and system-level measures.	Adequate staffing and appropriate mix. Optimise triage pathways and workflows for remote encounters.^35 36 48 65^ Protocol for times of extreme stress (staff absence, high demand). Reduce distractions.^19 20^ Optimise relational continuity for complex and vulnerable patients (eg, elderly) and continuity of illness episode for all patients.^41^ Provide training for all staff (not just in the technology); train for capability (taking initiative, playing hunches).^21 66^ Encourage workarounds and purposively develop norms for flexible working.^19^
Advice directed at patients and carers.	Think about how to describe your symptoms clearly before the appointment (write down key points if that helps you). Think about whether you need to have someone with you when you have your remote appointment (eg, to help with the technology or with communication). If you think an in-person consultation is needed, say so when you book the appointment and explain why. An in-person appointment is likely to be needed for: Chest pain/shortness of breath. Abdominal pain. Injury caused by a fall or accident. Unusual lump. Urgent mental health problem. Persistent skin problem. A child or someone in care who is unwell. If you have already had two remote appointments for a problem that is not improving. Be sure to tell the clinician all the key points about the current problem, even if you have told someone else from the surgery beforehand. Mention other conditions that may be relevant—for example, diabetes, a heart or chest condition, or a mental health condition. If you are very concerned about the problem, especially if things are getting worse, say so clearly. Ask the clinician to explain what happens next after the appointment and what to do if your symptoms do not improve. If you would like them to explain something again (to you or the person helping you), ask. Ask them to send you instructions (eg, via text message) if you would like this, and to include any further information such as a leaflet.

### Unanswered questions and future research

This study has helped explain where the key risks lie in remote primary care encounters, which in our dataset were almost all by telephone. It has revealed examples of how front-line staff create and maintain a safety culture, thereby helping to prevent such incidents. We suggest four key avenues for further research. First, additional ethnographic studies in general practice might extend these findings and focus on specific subquestions (eg, how practices identify, capture and learn from near-miss incidents). Second, ethnographic studies of out-of-hours services, which are mostly telephone by default, may reveal additional elements of safety culture from which in-hours general practice could learn. Third, the rise in asynchronous e-consultations (in which patients complete an online template and receive a response by email) raises questions about the safety of this new modality which could be explored in mixed-methods studies including quantitative analysis of what kinds of conditions these consultations cover and qualitative analysis of the content and dynamics of the interaction. Finally, our findings suggest that the safety of new clinically related ‘assistant’ roles in general practice should be urgently evaluated, especially when such staff are undertaking remote assessment or remote triage.

## Data Availability

Data are available upon reasonable request. Details of real safety incidents are not available for patient confidentiality reasons. Requests for data on other aspects of the study from other researchers will be considered.

## References

[1] England . Advice on how to establish a remote ‘total triage’ model in general practice using online consultations. London NHS England; 2020. Available: https://madeinheene.hee.nhs.uk/Portals/0/Advice%20on%20how%20to%20establish%20a%20remote%20total%20triage%20model%20in%20GP%20using%20online%20consultations.pdf [Accessed 23 Mar 2023].

[2] Singh V , Sarbadhikari SN , Jacob AG , et al . Challenges in delivering primary care via telemedicine during COVID-19 pandemic in India: a review synthesis using systems approach. J Family Med Prim Care 2022;11:2581–8. 10.4103/jfmpc.jfmpc_1559_21 36119291 PMC9480770

[3] Hall Dykgraaf S , Desborough J , de Toca L , et al . A decade’s worth of work in a matter of days”: the journey to telehealth for the whole population in Australia. Int J Med Inform 2021;151. 10.1016/j.ijmedinf.2021.104483 PMC8103781 33984625

[4] Koonin LM , Hoots B , Tsang CA , et al . Tong I: trends in the use of telehealth during the emergence of the COVID-19 pandemic—United States, January–March 2020. MMWR Morb Mortal Wkly Rep 2020;69:1595–9. 10.15585/mmwr.mm6943a3 33119561 PMC7641006

[5] England NHS . Updated standard operating procedure (SOP) to support restoration of general practice services. London: NHS England; 2021. Available: https://www.england.nhs.uk/wp-content/uploads/2021/05/B0497-GP-access-letter-May-2021-FINAL.pdf [Accessed 16 Jun 2022].

[6] Mroz G , Papoutsi C , Greenhalgh T . UK newspapers ‘on the Warpath’: media analysis of general practice remote consulting in 2021. Br J Gen Pract 2022;72:e907–15. 10.3399/BJGP.2022.0258 36192357 PMC9550315

[7] Healthcare safety investigation branch: NHS 111’s response to callers with COVID-19-related symptoms during the pandemic. 2022. Available: https://www.hsib.org.uk/investigations-and-reports/response-of-nhs-111-to-the-covid-19-pandemic/nhs-111s-response-to-callers-with-covid-19-related-symptoms-during-the-pandemic [Accessed 25 Jun 2023].

[8] Mroz G , Papoutsi C , Greenhalgh T . From ‘A rapid and necessary revolution’ to‘Telemedicine killed the PE teacher’: changing representations of remote GP consultations in UK media during the COVID-19 pandemic. In: Lewis M , Govander E , Holland K , eds. communicating COVID-19. London Palgrave, 2022.

[9] Royal College of General Practitioners . Fit for the future? GP pressures 2023. London: RCGP, 2023.

[10] NHS Confederation . Health and care face operational and financial pressures like never before Leeds: NHS Confederation. 2023. Available: https://www.nhsconfed.org/articles/health-and-care-face-operational-and-financial-pressures-never#:~:text=But%20the%20NHS%20is%20currently,facing%20its%20largest%20financial%20deficit [Accessed 10 Oct 2023].

[11] Warner M , Zaranko B . Pressures on the NHS. IFS Green Budget Chapter 6. London: IFS, 2023.

[12] Royal College of General Practitioners . The future role of remote consultations & patient ‘triage. London: RCGP, 2021.

[13] Neves AL , Li E , Gupta PP , et al . Virtual primary care in high-income countries during the COVID-19 pandemic: policy responses and lessons for the future. Eur J Gen Pract 2021;27:241–7. 10.1080/13814788.2021.1965120 34431426 PMC8404680

[14] England NHS . London: NHS England; Report a Patient Safety Incident, July . 2023 Available: https://www.england.nhs.uk/patient-safety/report-patient-safety-incident

[15] Panagioti M , Khan K , Keers RN , et al . Prevalence, severity, and nature of preventable patient harm across medical care settings: systematic review and meta-analysis. BMJ 2019;366. 10.1136/bmj.l4185 PMC6939648 31315828

[16] Panesar SS , deSilva D , Carson-Stevens A , et al . How safe is primary care? A systematic review. BMJ Qual Saf 2016;25:544–53. 10.1136/bmjqs-2015-004178 26715764

[17] Campbell JL , Britten N , Green C , et al . The effectiveness and cost-effectiveness of telephone triage of patients requesting same day consultations in general practice: study protocol for a cluster randomised controlled trial comparing nurse-led and GP-led management systems (ESTEEM). Trials 2013;14:4. 10.1186/1745-6215-14-4 23286331 PMC3574027

[18] Giesen P , Smits M , Huibers L , et al . Quality of after-hours primary care in the Netherlands: a narrative review. Ann Intern Med 2011;155:108–13. 10.7326/0003-4819-155-2-201107190-00006 21768584

[19] Hollnagel E , Wears RL , Braithwaite J . From Safety-I to Safety-II: a white paper. The resilient health care net: published simultaneously by the University of Southern Denmark. Florida, USA: University of Florida, 2015.

[20] Institute of Medicine (US) . Crossing the quality chasm: a new health system for the 21st century. Washington, DC: National Academy Press, 2001. 25057539

[21] Jerak-Zuiderent S . Certain uncertainties: modes of patient safety in healthcare. Soc Stud Sci 2012;42:732–52. 10.1177/0306312712448122 23189612

[22] Greenhalgh T , Shaw SE , Alvarez Nishio A , et al . Protocol: remote care as the ‘new normal’? multi-site case study in UK general practice. NIHR Open Res 2022;2:46. 10.3310/nihropenres.13289.1 37881300 PMC10593351

[23] Greenhalgh T , Shaw SE , Alvarez Nishio A , et al . Remote care in UK general practice: baseline data on 11 case studies. NIHR Open Res 2022;2:47. 10.3310/nihropenres.13290.2 36814638 PMC7614213

[24] Greenhalgh T , Payne RE , Hemmings N , et al . Remote services in general practice: who needs to be trained in what, when and how? Br J Gen Pract 2023:BJGP. 10.3399/BJGP.2023.0251

[25] Braun V , Clarke V . Using thematic analysis in psychology. Qualitative Research in Psychology 2006;3:77–101. 10.1191/1478088706qp063oa

[26] Winter R . Fictional-critical writing: an approach to case study research by practitioners and for in-service and pre-service work with teachers. In: The enquiring teacher. edn. Routledge, 2013: 231–48.

[27] Greenhalgh T , Ladds E , Hughes G , et al . Why do GPs rarely do video consultations? qualitative study in UK general practice. Br J Gen Pract 2022;72:e351–60. 10.3399/BJGP.2021.0658 35256385 PMC8936181

[28] Lupton D , Maslen S . Telemedicine and the senses: a review. Sociology Health & Illness 2017;39:1557–71. 10.1111/1467-9566.12617 Available: https://onlinelibrary.wiley.com/toc/14679566/39/8 29071731

[29] Banks J , Farr M , Salisbury C , et al . Use of an electronic consultation system in primary care: a qualitative interview study. Br J Gen Pract 2018;68:e1–8. 10.3399/bjgp17X693509 29109115 PMC5737315

[30] Griese L , Berens E-M , Nowak P , et al . Challenges in navigating the health care system: development of an instrument measuring navigation health literacy. International Journal of Environmental Research and Public Health 2020;17:5731. 32784395 10.3390/ijerph17165731 PMC7460304

[31] Macdonald S , Blane D , Browne S , et al . Illness identity as an important component of candidacy: contrasting experiences of help-seeking and access to care in cancer and heart disease. Social Science & Medicine 2016;168:101–10. 10.1016/j.socscimed.2016.08.022 27643844

[32] Edwards PJ , Bennett-Britton I , Ridd MJ , et al . Factors affecting the documentation of spoken safety-netting advice in routine GP consultations: a cross-sectional study. Br J Gen Pract 2021;71:e869–76. 10.3399/BJGP.2021.0195 34489251 PMC8436774

[33] Brooks W , Smith K , Warren C , et al . Safety netting in the COVID-19 clinical assessment service. Br J Gen Pract 2021;71:541–2. 10.3399/bjgp21X717773 PMC8686412 34824083

[34] Smith K , Brooks W , Challiner J , et al . Quality assurance of remote clinical assessments in the NHS. Lancet 2021;398. 10.1016/S0140-6736(21)02328-X PMC8616570 34838172

[35] Rosen R , Wieringa S , Greenhalgh T , et al . Clinical risk in remote consultations in general practice: findings from in-COVID-19 pandemic qualitative research. BJGP Open 2022;6. 10.3399/BJGPO.2021.0204 PMC9680756 35487581

[36] Wieringa S , Neves AL , Rushforth A , et al . Safety implications of remote assessments for suspected COVID-19: qualitative study in UK primary care. BMJ Qual Saf 2023;32:732–41. 10.1136/bmjqs-2021-013305 PMC8927927 35260414

[37] Bolton R , Logan C , Gittell JH . Revisiting relational coordination: a systematic review. The Journal of Applied Behavioral Science 2021;57:290–322. 10.1177/0021886321991597

[38] Edmondson AC , Lei Z . Psychological safety: the history, Renaissance, and future of an interpersonal construct. Annu Rev Organ Psychol Organ Behav 2014;1:23–43. 10.1146/annurev-orgpsych-031413-091305 Available: https://www.annualreviews.org/toc/orgpsych/1/1

[39] Leone E , Eddison N , Healy A , et al . Do UK Allied health professionals (Ahps) have sufficient guidelines and training to provide Telehealth patient consultations Hum Resour Health 2022;20:82. 10.1186/s12960-022-00778-1 36471340 PMC9721053

[40] Dakin F , Rai T , Paparini S , et al . Supporting your support staff during crises: tipe for creating a relational workplace. BMJ Leader 2023.

[41] Ladds E , Greenhalgh T , Byng R , et al . A contemporary ontology of continuity in general practice: capturing its multiple essences in a Digital age. Soc Sci Med 2023;332. 10.1016/j.socscimed.2023.116112 37535988

[42] Ladds E , Khan M , Moore L , et al . How have remote care approaches impacted continuity? A mixed-studies systematic review. Brit J Gen Pract 2023. 10.3399/BJGP.2022.0398 Available: 10.3399/BJGP.2022.0398 PMC10058181 37105731

[43] Ladds E , Greenhalgh T . Modernising continuity: a new conceptual framework. Br J Gen Pract 2023;73:246–8. 10.3399/bjgp23X732897 37230773 PMC10229150

[44] McKinstry B , Hammersley V , Burton C , et al . The quality, safety and content of telephone and face-to-face consultations: a comparative study. Qual Saf Health Care 2010;19:298–303. 10.1136/qshc.2008.027763 20430933

[45] Hewitt H , Gafaranga J , McKinstry B . Comparison of face-to-face and telephone consultations in primary care: qualitative analysis. Br J Gen Pract 2010;60:e201–12. 10.3399/bjgp10X501831 20423575 PMC2858552

[46] Shaw SE , Seuren LM , Wherton J , et al . Video consultations between patients and clinicians in diabetes, cancer, and heart failure services: linguistic Ethnographic study of Video-mediated interaction. J Med Internet Res 2020;22:e18378. 10.2196/18378 32391799 PMC7248806

[47] Hammersley V , Donaghy E , Parker R , et al . Comparing the content and quality of video, telephone, and face-to-face consultations: a non-randomised, quasi-experimental, exploratory study in UK primary care. Br J Gen Pract 2019;69:e595–604. 10.3399/bjgp19X704573 31262846 PMC6607843

[48] Khan MNB . Telephone consultations in primary care, how to improve their safety, effectiveness and quality. BMJ Qual Improv Rep 2013;2. 10.1136/bmjquality.u202013.w1227 PMC4652702 26734172

[49] Sharma SC , Sharma S , Thakker A , et al . Revolution in UK general practice due to COVID-19 pandemic: a cross-sectional survey. Cureus 2020;12:e9573. 10.7759/cureus.9573 32913690 PMC7474564

[50] Johnsen TM , Norberg BL , Kristiansen E , et al . Suitability of Video consultations during the COVID-19 pandemic Lockdown: cross-sectional survey among Norwegian general practitioners. J Med Internet Res 2021;23:e26433. 10.2196/26433 33465037 PMC7872327

[51] Seuren LM , Wherton J , Greenhalgh T , et al . Physical examinations via Video for patients with heart failure: qualitative study using conversation analysis. J Med Internet Res 2020;22:e16694. 10.2196/16694 32130133 PMC7059096

[52] Giesen P , Ferwerda R , Tijssen R , et al . Safety of telephone triage in general practitioner cooperatives: do triage nurses correctly estimate urgency Qual Saf Health Care 2007;16:181–4. 10.1136/qshc.2006.018846 17545343 PMC2465002

[53] NHS Resolution . An overview of the first year of the clinical negligence scheme for general practice (CNSGP). London: NHS Resolution, 2022.

[54] Marincowitz C , Stone T , Bath P , et al . Accuracy of telephone triage for predicting adverse outcomes in suspected COVID-19: an observational cohort study linking NHS 111 telephone triage, primary and secondary Healthcare and mortality records. IJPDS 2022;7. 10.23889/ijpds.v7i3.1777 PMC8983415 35354665

[55] Antonio MG , Williamson A , Kameswaran V , et al . Targeting patients’ cognitive load for telehealth video visits through student-delivered helping sessions at a United States federally qualified health center: equity-focused, mixed methods pilot intervention study. J Med Internet Res 2023;25:e42586. 10.2196/42586 36525332 PMC9897309

[56] Oudshoorn N . Diagnosis at a distance: the invisible work of patients and healthcare professionals in cardiac telemonitoring technology. Sociology Health & Illness 2008;30:272–88. 10.1111/j.1467-9566.2007.01032.x Available: https://onlinelibrary.wiley.com/toc/14679566/30/2 18290936

[57] Mehmi A , Winters D , Newman T , et al . Remote clinics: a panacea during the pandemic or a promoter of health inequality Br J Hosp Med 2020;81:1–3. 10.12968/hmed.2020.0555 33135918

[58] Huibers L , Smits M , Renaud V , et al . Safety of telephone triage in out-of-hours care: a systematic review. Scand J Prim Health Care 2011;29:198–209. 10.3109/02813432.2011.629150 22126218 PMC3308461

[59] Remote consultations. n.d. Available: https://www.gmc-uk.org/ethical-guidance/ethical-hub/remote-consultations

[60] Doherty M , Neilson S , O’Sullivan J , et al . Barriers to Healthcare and self-reported adverse outcomes for autistic adults: a cross-sectional study. BMJ Open 2022;12:e056904. 10.1136/bmjopen-2021-056904 PMC8883251 35193921

[61] Husain L , Greenhalgh T , Hughes G , et al . Desperately seeking Intersectionality in Digital health disparity research: narrative review to inform a richer Theorization of multiple disadvantage. J Med Internet Res 2022;24:e42358. 10.2196/42358 36383632 PMC9773024

[62] Carson-Stevens A , Hibbert P , Williams H , et al . Characterising the nature of primary care patient safety incident reports in the England and Wales national reporting and learning system: a mixed-methods agenda-setting study for general practice. Health Serv Deliv Res 2016;4:1–76. 10.3310/hsdr04270 27656729

[63] Rees P , Edwards A , Powell C , et al . Patient safety incidents involving sick children in primary care in England and Wales: a mixed methods analysis. PLoS Med 2017;14:e1002217. 10.1371/journal.pmed.1002217 28095408 PMC5240916

[64] Morris RL , Giles S , Campbell S . Involving patients and Carers in patient safety in primary care: A qualitative study of a Co‐Designed patient safety guide. Health Expect 2023;26:630–9. 10.1111/hex.13673 36645147 PMC10010084

[65] King K SM . Video consulting, not just a consultation plus Tech. BJGP Live, 2020.

[66] Fraser SW , Greenhalgh T . Greenhalgh T: coping with complexity: educating for capability. BMJ 2001;323:799–803. 10.1136/bmj.323.7316.799 11588088 PMC1121342

